# Long‐term annual and spatial variation of polygyny in the white‐throated dipper (*Cinclus cinclus*)

**DOI:** 10.1002/ece3.9416

**Published:** 2022-10-22

**Authors:** Bjørn Walseng, Joël M. Durant, Dag O. Hessen, Kurt Jerstad, Anna L. K. Nilsson, Ole W. Røstad, Tore Slagsvold

**Affiliations:** ^1^ Norwegian Institute for Nature Research Oslo Norway; ^2^ Department of Biosciences University of Oslo Oslo Norway; ^3^ Jerstad Viltforvaltning Mandal Norway; ^4^ Norwegian Institute for Nature Research Bergen Norway; ^5^ Department of Ecology and Natural Resource Management Norwegian University of Life Sciences Ås Norway

**Keywords:** frequency of polygyny, mating strategy, monogamous, territory quality, white‐throated dipper

## Abstract

Mating strategies are key components in the fitness of organisms, and notably in birds the occurrence of monogamy versus polygyny has attracted wide interest. We address this by a very comprehensive dataset (2899 breeding events spanning the years 1978–2019) of the white‐throated dipper *Cinclus cinclus*. Though the mating system of this species has been regarded as generally monogamous, we find that 7% of all breeding events were performed by polygynous males (approximately 15% of all pairs). The fraction of polygyny has been stable over the entire study period irrespective of population size. The assumption that polygyny is most common at low population density was not supported. Surprisingly, there was no difference between polygynous and monogamous males with regard to the quality of the territories they inhabited, ranked according to their overall frequency of use. The most common age group, first‐year breeders, dominated among monogamous males, while among polygynous males second‐year breeders were most common, followed by third and first‐year breeders. The primary females were in general older than females mated to monogamous males, also when controlled for their general frequency in the population. The majority of the two females mated to a polygynous male, bred in the vicinity of each other. The probability for a male to be involved in polygyny more than once, was significantly higher than by chance, suggesting phenotypic quality differences among males.

## Graphical abstract

Mating strategies, and the role and prevalence of polygyny is a core topic in behavioral ecology. With a quite extraordinary time‐series and dataset on dippers at hand, we assess both the years with different climatic impacts and widely different breeding populations as well as a large spatial frequency and key determinants of polygyny. Despite often perceived as a typically monogamous species, we found that breeding events performed by polygamous males, involved approximately 15% of all pairs.

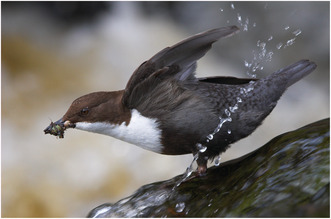

## 1 INTRODUCTION

The animal kingdom possesses a tremendous variability in reproductive strategies, yet “all roads lead to Rome” in the sense that all strategies ultimately aim to maximizing gene transfer to the next generations (Kokko & Jennions, 2008). Among metazoans, sexual reproduction is the norm, with different variants of mono‐ or polygamy. From the male's perspective, monogamy can promote reproductive fitness by parental care, or alternatively, the male can reproduce with more than one female spending less or no effort on parental care, defined as polygyny.

Detailed observations of the behavior of each member of a population in the wild are needed to reveal the true mating system of a species (Davies, 1992). Notably, in birds, the occurrence of polygyny has attracted wide interest and while social monogamy is most common, there is also a number of polygynous species (Cockburn, 2006; Lack, 1968). However, in species assumed to be monogamous, like territorial birds where both sexes provide parental care, there may nevertheless be some males adopting a polygynous mating strategy. In primarily monogamous species, polygyny might be an opportunistic strategy used by any male if chance allows, or strategy possessed by certain, likely dominant, individuals (Slagsvold et al., 1988).

Initially, the occurrence of polygyny was mainly judged from a male's perspective, where the trait was assumed to have evolved when female reproductive success is not totally dependent on full male assistance (Trivers, 1972). Later on, this was combined with a female's perspective, resulting in a better understanding of the evolution of mating systems in general (Orians, 1969). It was recognized that in animals with biparental care, a classic conflict of interest may exist between the sexes when the males benefit from attracting more than one female, whereas the females benefit from monopolizing male parental care.

The polygyny threshold model says that polygyny may occur when the difference in breeding situation quality (territory and male quality) between the two males is so great in favor of the already‐mated male that it compensates for the expected costs of polygyny caused by reduction in male parental care to the secondary female(s) (Orians, 1969). However, prospecting females may not always have an easy choice, depending on a number of factors, including the local sex ratio and variation in the quality of the male, his territory and the nest sites (Canal et al., 2021). For example, in blue tits *Cyanistes caeruleus*, a female may accept to mate with an already‐mated male because she has previous experience from the local area and thus from his territory, or because she has recently lost her own mate from predation after nest building has started (Kempenaers, 1994). Notably, for migratory species, female arrival time may also be crucial, as late females may have few options to find unpaired males. Costs of searching for a mate and nest site may also be important in general, as shown in the pied flycatcher *Ficedula hypoleuca* (Slagsvold et al., 1988).

Typically, in secondary cavity nesting passerine birds, such as in the pied flycatcher, suitable nest sites are often few and females may accept to settle with an already‐mated male if he is able to provide a good site (Lundberg & Alatalo, 1992). In this species, a male may try to attract females to territories often located several hundred meters apart with conspecific birds occupying territories in between (Slagsvold & Lifjeld, 1994). It has been suggested that the already‐mated males displaying in their secondary territory thereby deceive prospecting females about their mating status (Alatalo et al., 1981). However, recent studies have shown that the prospecting females are aware of male mating status but may settle with an already‐mated male because of lack of better alternatives (Dale & Slagsvold, 1996). The males may display far away from the first territory to escape aggression from the first female (Slagsvold et al., 1999) because females are able to locate their own mate from at long distances from his song (Lampe & Slagsvold, 1998). In this species, and in many other birds with biparental care, the secondary females may suffer reduced breeding success, yet it may still pay off to attempt nesting if the alternative is not breeding at all (Kempenaers, 1994; Nilsson et al., 2020; Slagsvold & Lifjeld, 1994).

**FIGURE 1:**
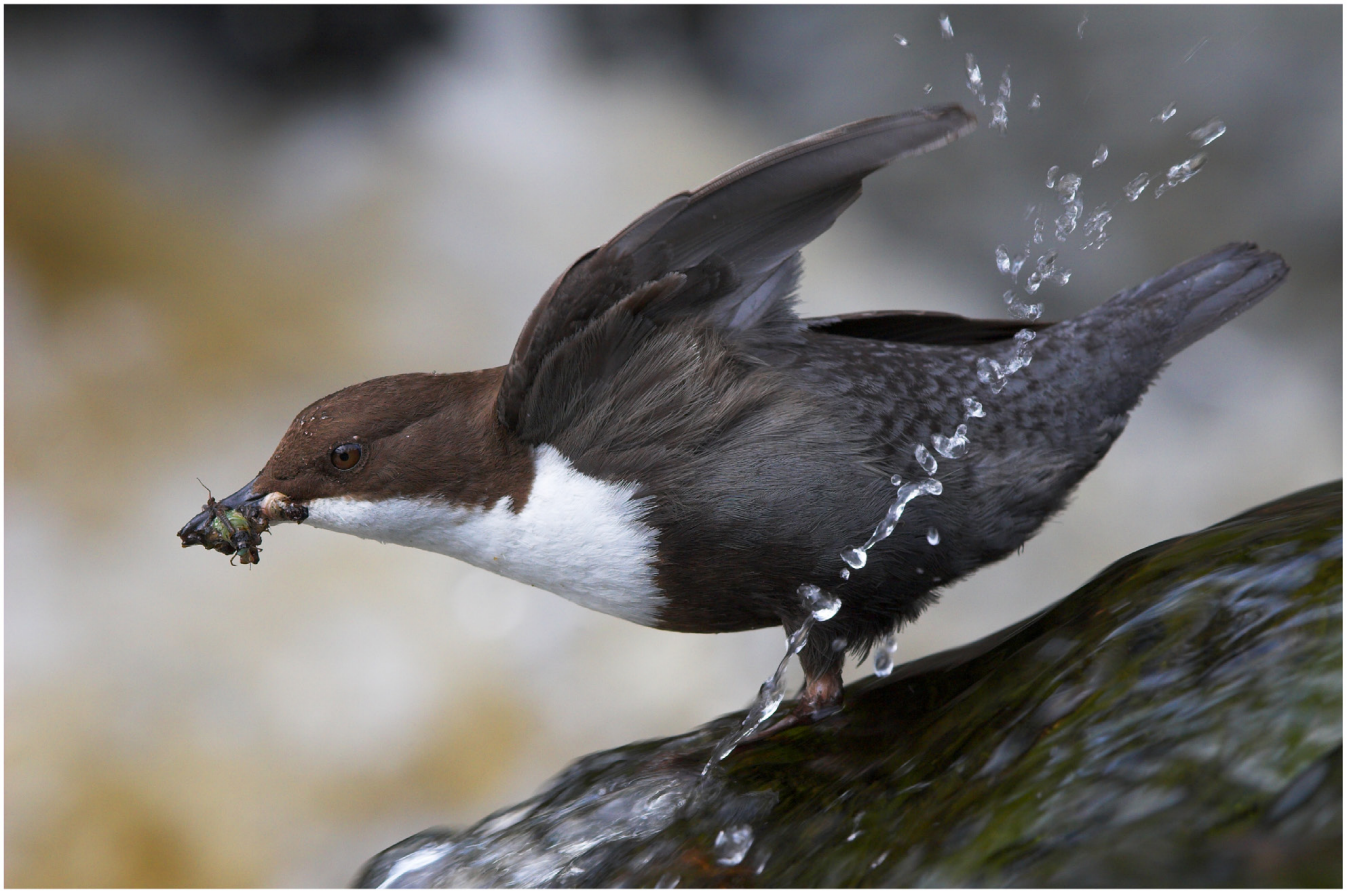
The dipper (photo: Geir Rune Løvstad).

Most long‐term studies of facultative polygyny in birds have been on secondary hole‐nesting passerines that have been provided with nest boxes. In this study, we take advantage of an unusually large dataset on breeding in the white‐throated dipper *Cinclus cinclus* (Figure 1, termed the dipper hereafter) to explore facultative polygyny. Both the dataset per se and the species with its nesting confined along the rivers and tributaries within a specified catchment, allows for robust testing of breeding strategies and incidence of polygyny. The dipper species is monomorphic and mainly socially monogamous, and both sexes take part in nest‐building and parental care. The proportion of offspring sired by extra‐pair copulations seems to be low (Øigarden et al., 2010). It has been documented that dipper males can pair with two or three females (K. Jerstad, unpublished data; Wilson, 1996). In contrast to most other bird species, the territory of the dipper has a linear spatial distribution related to waterways. Male dippers defend a part of a river system, and to attract more than one female the males need to provide two or more suitable breeding localities. The dipper's nest is almost always located near rapids, probably to escape predation. The number of suitable nest sites within a territory may therefore be restricted, especially in slow‐flowing parts of the river. Thus, the availability of suitable nest sites may restrict polygyny but promote its occurrence because females may also have difficulty finding good nest sites defended by unmated males. Thus, also a high number of females relative to males may promote polygyny (Emlen & Oring, 1977). Also, additional important factors according to the polygyny threshold model are whether females are able to reproduce with limited male assistance (Orians & Pearson, 1969), and whether a second female is allowed to settle on the territory by the first female (Kempenaers, 1994; Slagsvold et al., 1992).

In the dipper, all activities are linked to water, and the birds can easily be captured and banded. Thus, an accurate census of the breeding population, and details of the mating system can be obtained. In our unique dataset in terms of spatial and temporal coverage, the dipper population has been surveyed continuously since 1978 and the adults have been banded with color bands and numbered metal bands, the young's with metal bands. The data thus offer an excellent opportunity to address how various drivers and strategies affect population size and reproductive strategies spatially and temporally. The size of the dipper population has varied annually in an approximately 8‐year cycle which seemed to correlate well with the semi‐regular variation in climate as assessed by the NAO‐index (Nilsson et al., 2011; Sæther et al., 2000).

Though the knowledge of the breeding ecology in dippers is comprehensive, there are still questions that remain to be answered regarding polygyny. According to Wilson (1996): “The dipper is a species in which the incidence of polygyny apparently varies both between populations and between years within populations, and further work is needed to understand the factors controlling this variation.” With an exceptionally large dataset at hand, covering many years with different climatic impacts and widely different breeding populations, we are well equipped to assess both the frequency and key determinants of polygyny.

The aim of the present study was first to test for the prevalence of polygyny in the local dipper population, and then assess whether the prevalence of this trait varied in relation to variables such as total population size, male and female age, suitable nest sites within territories, the distances between neighboring male territories, and the nesting phenology (onset of breeding). Because the population size may vary six‐fold among years, we hypothesize that the density of the breeding population will affect the frequency of polygyny, being most common in years with low density when males can defend a large territory with potentially more nesting sites and potentially less conflict among a reduced number of females. We also expect more older than yearling males to be polygynous because they have more experience (Potti & Montalvo, 1993; Santoro et al., 2022). However, the rate of senescence may be quite high in small passerines (Holand et al., 2016), and so we also asked whether a decline existed in frequency of polygyny at older ages. Males providing better breeding sites (judged from frequency of use) are supposed to be polygynous more often. If the probability of polygyny is related to breeding site quality and/or male quality, we expect the likelihood for a male to be polygynous to be positively correlated across years. Finally, males occupying territories where egg‐laying started early, would allow more time for the male to display and attract more females. A full analysis of potential fitness gains requires more extensive evaluation also of female and offspring success and is beyond the scope of this paper.

## 2 MATERIALS AND METHODS

### 2.1 Study area

**FIGURE 2:**
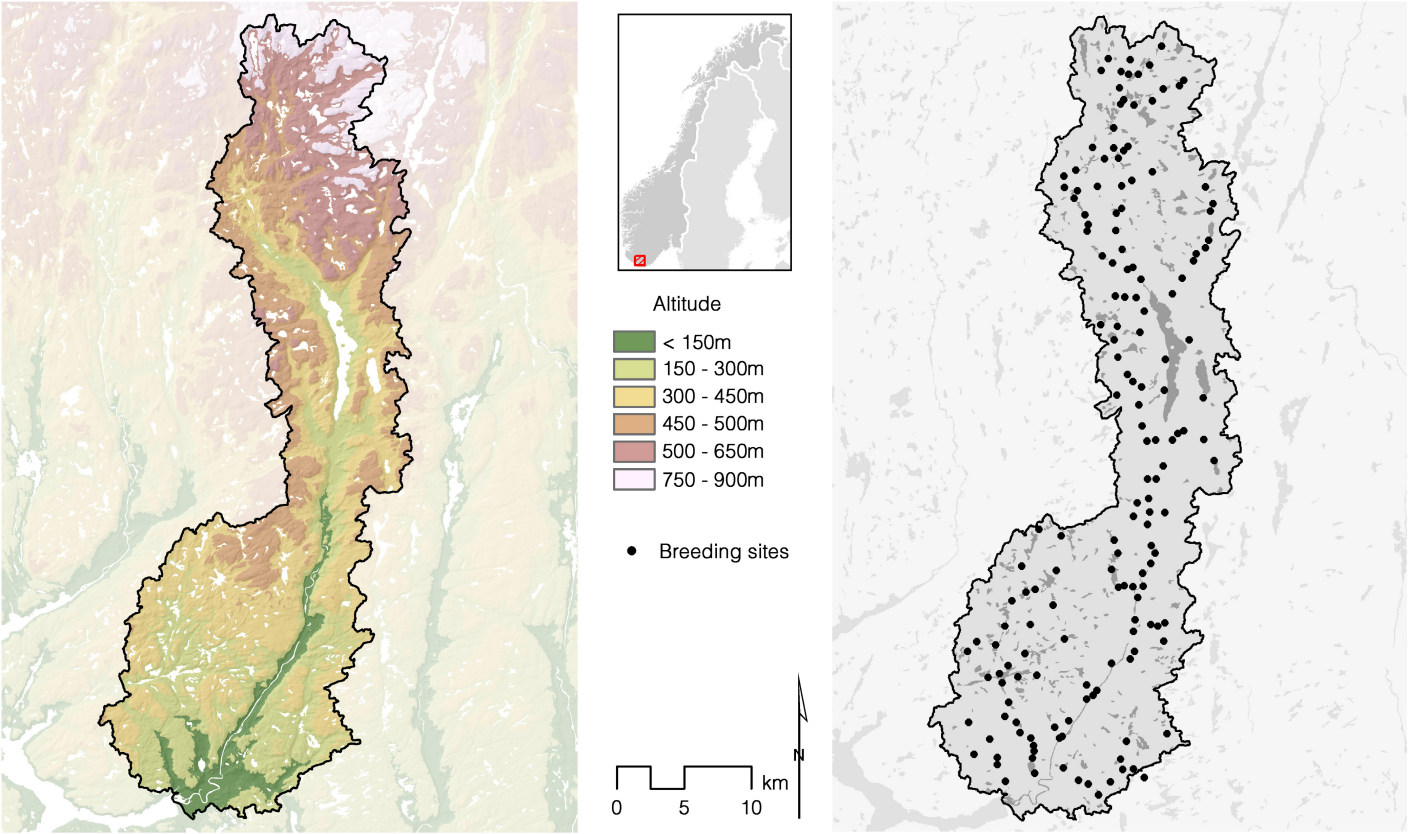
The River Lygna watershed (left). The map to the right shows the location of all breeding sites (*n* = 165).

The fieldwork was conducted in the river system of Lygna, southern Norway (58°08′–58°40′N, 6°56′–7°20′E), that ranges from sea‐level to an altitude of 966 m (Figure 2). The river Lygna, oriented north–south, and with a catchment area of about 680 km^2^, has cut into the surrounding undulating plain at 200–400 m above sea level (a.s.l.) in the south and 500–700 m a.s.l. in the north. A high number of tributaries of different sizes drain water down the hillsides before entering the main river. In the middle of the river system, the river is interrupted by the Lake Lygne (surface area 7.56 km^2^). The entire catchment and river system is not utilized for hydropower and it is protected against further development.

### 2.2 Material

The fieldwork was standardized and started in 1978, when virtually all possible breeding sites in the river system were mapped and regularly visited, and is still ongoing, whereas the present study only covers the time series until 2019. Pairs of dippers defend a territory during the breeding season, which typically is initiated in the beginning of March and ends in mid‐July depending on elevation. We visited all known breeding sites in the morning hours during the nest building season and scanned for dipper activity. Within their territories, dippers build a nest which consists of an outer layer of mosses, which can be used for several consecutive years, and an inner lining which is renewed for each breeding attempt. A breeding attempt was defined as a nest with a completed lining of leaves.

The total number of available breeding sites that have been occupied or where breeding attempts have been recorded in the river system of Lygna is 165 (Figure 2), ranging between 20 and 117 annually. In the many steep tributaries draining to the main river, nest sites may be found widely distributed along the tributaries. These tributary territories neighboring the main river also include a stretch of the main river for foraging. When nest sites are found in the main river or in larger tributaries, they are most often located in close proximity to freshwater rapids, and therefore concentrated within a restricted area. We have defined the most used nest site as the center for the territory. A neighbor territory is defined when it has been used in the same year. It means that new territories may arise if nesting should occur between two established sites used in the same year. Mapping the exact “border” between two territories would have been extremely time consuming and not necessary for our purpose. The number of nest sites that have been in use within a breeding site, has varied between 1 and 15. A total of 860 different nest sites have been recorded, located from 9 to 697 m a.s.l.

Dippers lay one egg per day, typically with a clutch size of 4–6 eggs. The female starts the incubation period, lasting approximately 17 days, after the clutch is complete, and there are usually <12 h elapsing between the hatching of the first and last egg (Borgström, 1991). Except for a few, rare observations of males taking part (i.e., Balat, 1964), the female dipper alone incubates (i.e., Tyler & Ormerod, 1994). The nestling period is about 22 days and both parents feed the young (Shaw, 1978).

The population size in a given breeding season is defined as the number of breeding females. A total of 3452 breeding attempts have been recorded over the entire study period (1978–2019). By use of color bands for individual identification, the identities of 3250 males and 3256 females have been recorded over this period. We define a female as secondary if she mated the same male, but initiated egg laying later than the primary female.

We also monitored male dippers and their nests in the catchments adjacent to River Lygna to assess whether a ringed male in River Lygna also had nested in another river. If so, this male was defined as polygynous and included in the analysis. More than 2000 nesting attempts have been recorded in the surrounding catchments (River Mandalselva, River Audna, and River Kvina) over the entire study period. We therefore assume that most of the polygynous males in the River Lygna were identified. Twenty‐nine males with failed nesting and then renesting with a different female in the same season, were assigned as monogamous.

In the present study area, an unknown fraction of the dipper population is partially migratory during winter. However, the wintering strategies of the dipper population of River Lygna do not seem to be related to age or sex (Poslinska, 2014), and should thus not affect our conclusions on the occurrence of polygyny.

### 2.3 Data analysis

**FIGURE 3:**
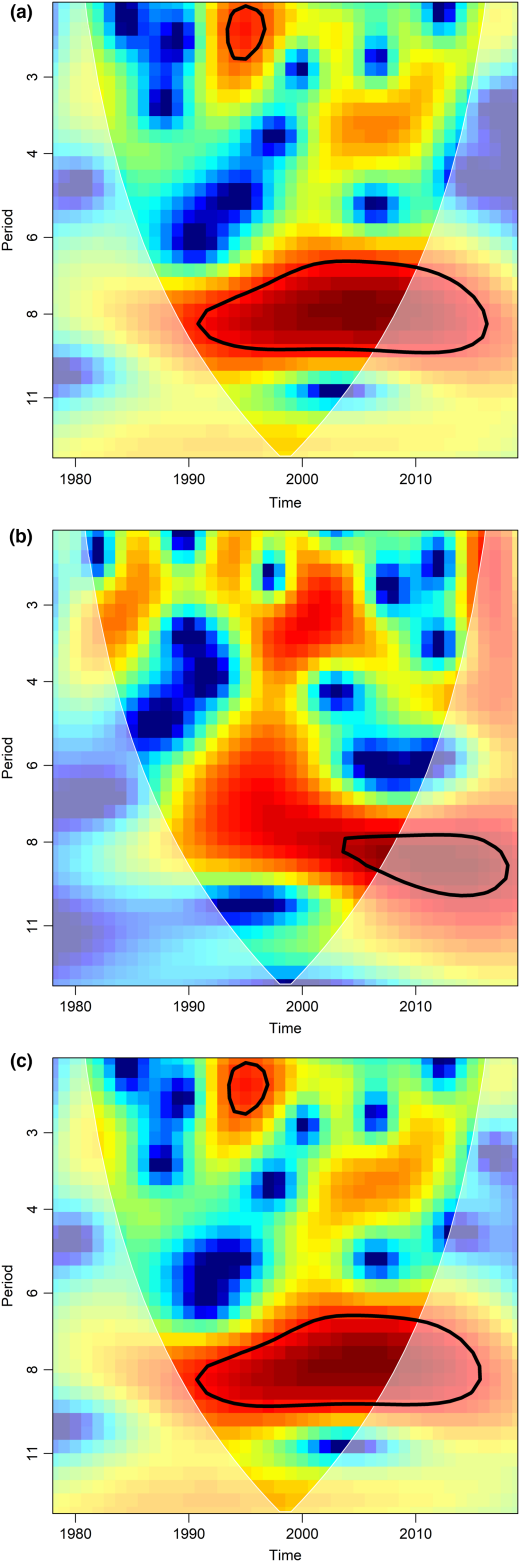
Wavelets analyses (using R package “*biwavelet*”) of the dipper breeding population size between 1978 and 2019 [(a) total breeding males, (b) only polygynous, (c) only monogamous]. The plots are the bias‐corrected power normalized by the variance (using “type = power.corr.norm”). The cone of influence (white lines) illustrates the loss in statistical power near the start and the end of the series and must be interpreted with caution. The red color, indicates that over time exists a dominant ca 8‐year periodicity covering the studied period. While not significant over the whole period (black line, 1991–2018), partly due to border effect, it is consistent for all categories of males.

To test whether there was a temporal pattern or cyclicity in the breeding number time series (i.e., whether a breeding numbers occurred regularly over the years), a wavelet analysis was applied. Wavelet analysis decomposes the time‐series into time and frequency domains and determines the dominant (significant) mode of variability (Cazelles et al., 2008). We used wavelet decomposition analysis from the package “biwavelet” in R (version 4.0.2) R Core Team (2020). The analysis can be displayed as 3D contour plots with breeding frequency along time (year) and at which frequency (cycle period in number of years) a fluctuation is significant (cf. Figure 3). The analysis was run for total males, polygonous males, and monogamous males separately.

**FIGURE 4:**
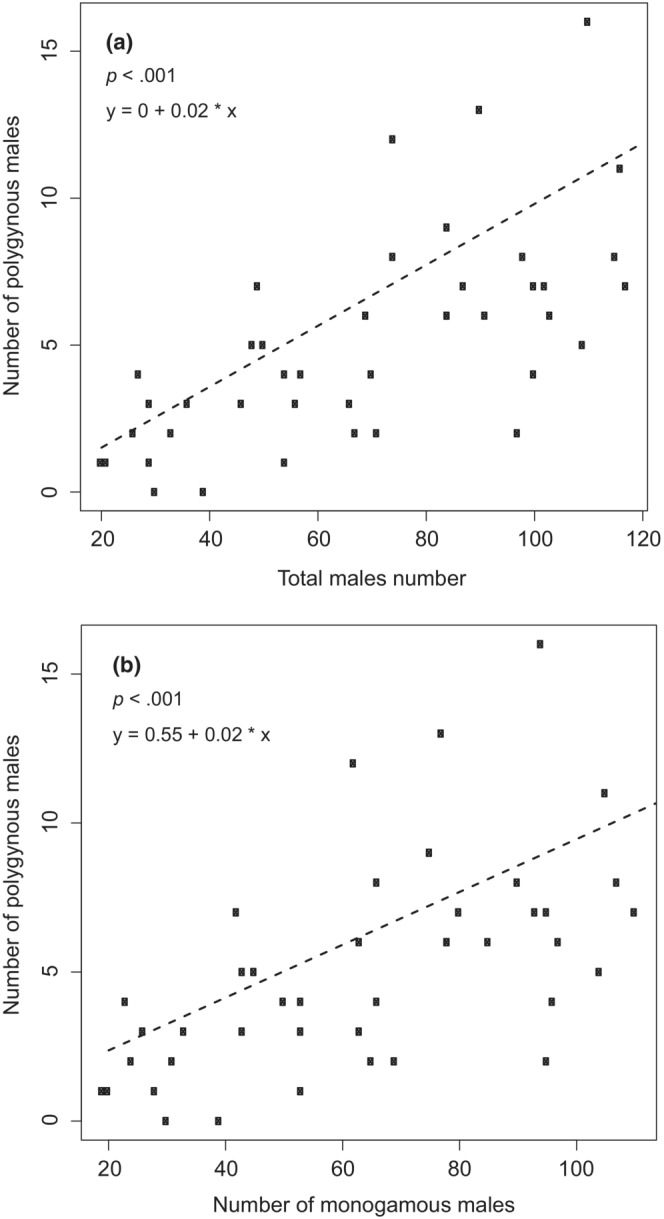
The number of polygynous dippers versus total population size (left) and number of monogamous males (right) over 42 years. Linear regression is the dotted line. *p* < .001 for both, with *R* ^2^ = .38 and .31, respectively.

The relationship between the number of polygynous male number to the total number of males (Figure 4a) and that of monogamous males (Figure 4b) was estimated using generalized linear models with the function *glm* in R. To take into account the overdispersion of the residuals, we used a *quasibinomial* error distribution. We did not detect any significant autocorrelation in the residuals (using autocorrelation function *acf*).

The description of the effect of age on polygamy/monogamy was done using the function *boxplot*. The significance difference between groups was estimated using a rank test (Siegel & Castellan, 1988) followed when necessary by a Dunn's test (1964) with the Benjamini–Hochberg *p*‐value adjustment (Benjamini & Hochberg, 1995), to report the differences among multiple pairwise comparisons, using the package *dunn.test* in R. The statistical tests are two‐tailed with an *α*‐level of .05.

## 3 RESULTS

### 3.1 Males

Of a total of 2899 breeding events over the period 1978–2019, 214 were performed by polygynous males (7.4%). The total number of breeding attempts varied annually between 20 and 117 (1978–2019), whereas the corresponding numbers of polygynous males ranged from 0 (in 1986 and 2012) to 17 (in 2017). In the 2 years without polygyny, the total population was low, consisting of only 33 and 29 pairs, respectively. The mean number of total breeding attempts and breeding attempts performed by monogamous and polygynous males were 67 and 5, respectively. Only once we could ascertain a male that remained unmated.

The number of polygynous males varied with the total number of nesting events, and as shown by wavelets analyses (Figure 3), the significant contours were approximately the same for the total population and for the monogamous males, suggesting 8 years cycles. If there had been a different pattern for the polygynous males, it would have affected the relationship between monogamous males and the total population. The only cycle that seemed significant for polygynous males was also approximately 8 years, being evident for the recent years as a result of more data.

There was a linear relationship between the total population of males and the number of polygynous males (Figure 4), as well as between the number of monogamous and polygynous males. Of 214 polygynous breeding attempts, 208 (97%) were performed with two females. The remaining six breeding attempts involved three females. Five of the polygynous males were breeding with one of the females in a territory situated outside River Lygna.

**FIGURE 5:**
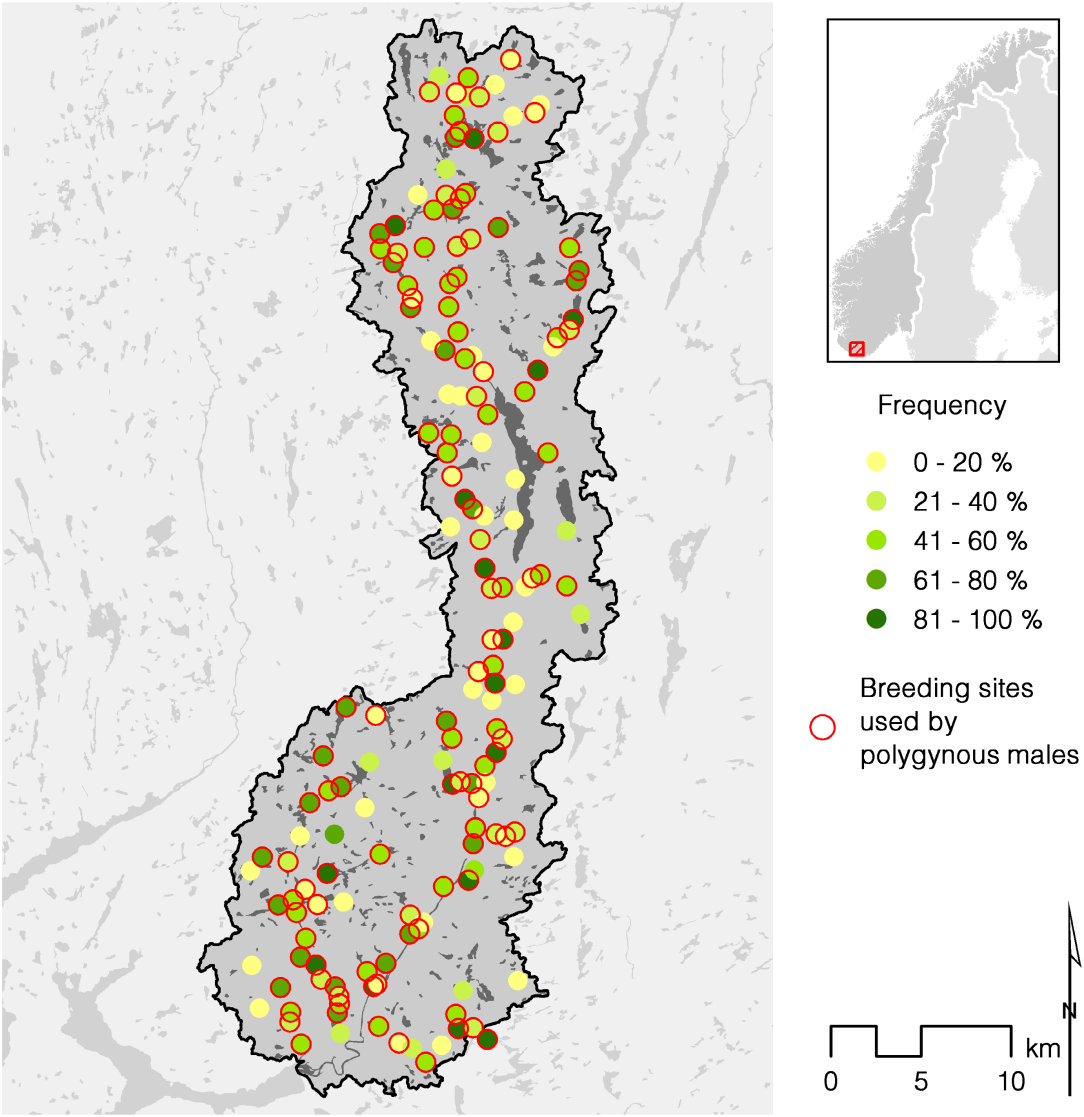
The frequency of use of breeding sites (% occupied of all years) in the river Lygna. Breeding sites used by polygynous males are encircled with a red ring. For distribution of elevation, see Figure 2.

**FIGURE 6:**
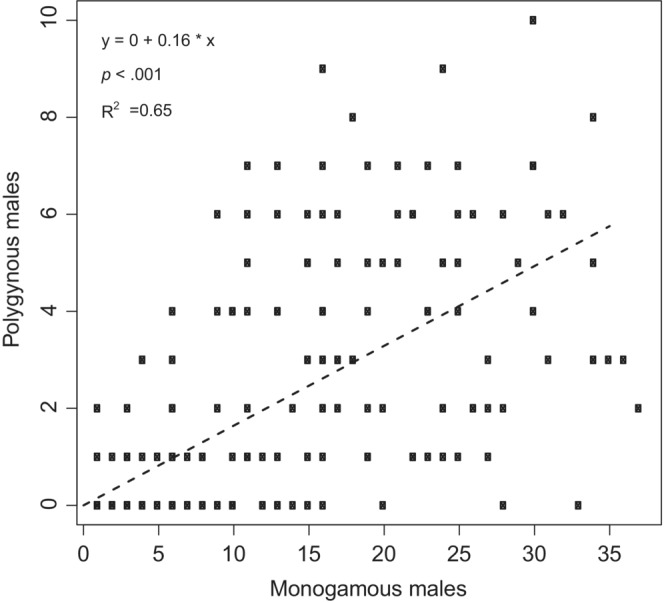
Number of years each breeding site (*n* = 165) has been in use by monogamous and polygynous males, respectively.

Dipper use of breeding sites varied from one that was used every year, to eight that were only used once during the entire study period. Polygyny has been recorded in a total of 120 (73%, *n* = 165) of the breeding sites (Figure 5). While 46 breeding sites were only used once (26) or twice (20), one breeding site was occupied by polygynous males for 10 years. However, we found no preference for polygynous versus monogamous males in specific breeding sites (*b* = 2.0, *df* = 163, *t* = 7.8, *p* < .001; Figure 6).

**FIGURE 7:**
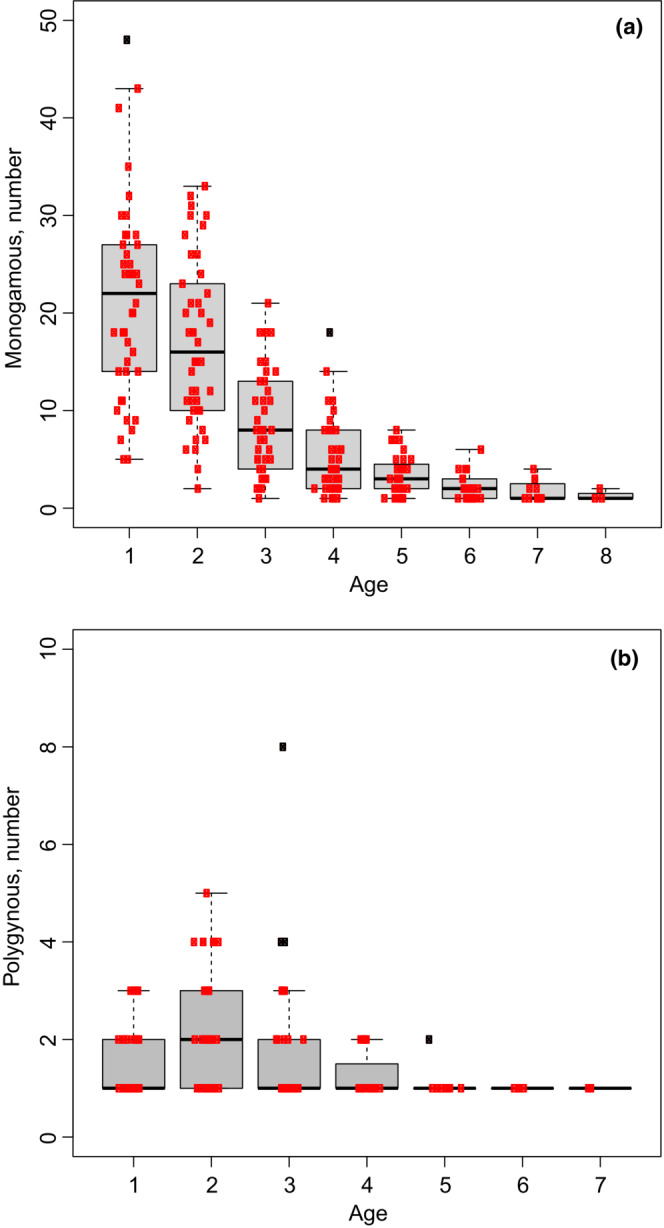
Age distribution summed per year of monogamous (left panel) and polygynous males (right panel), respectively. Each box is the summary of all the years (1 year is one point). The horizontal line is the median value, the box delimits the 1st and 3rd quartiles [interquartile range (IQR) criterion], while the whiskers (error bars) show the 5th and 95th percentiles. Black points are outliers. The dots are the data (in gray when outliers).

The mean ages of monogamous and polygynous males were 1.81 (*n* = 2070) and 2.49 years (*n* = 204), respectively. The most abundant age in the monogamous males was 1 year followed by increasing age classes (Figure 7, KW test *p* < .001, Dunn's test, *p* < .001). By comparison, the most abundant age for polygynous males was 2 years followed by third‐ and first‐year breeders (KW test, *p* < .001, Dunn's test, *p* < .05). Compared with monogamous males (see Figure 7) there was a higher fraction of 4‐ and 5‐year breeders among polygynous males. However, among the oldest breeders, 6‐ to 7‐year‐old monogamous males constituted 3% of the total breeding population, compared with 2% for the polygynous. The maximum breeding age for males was 8 years, recorded for four birds that were consistently socially monogamous.

Altogether 168 males were involved in 214 polygynous breeding events. Thirty of these males (18%) were polygynous for more than 1 year, and one of these males was in fact breeding with the same two females successively in 4 years. The probability for a polygynous male to be involved in polygyny more than once was significantly higher than by chance (7%) (*t*‐test, *p* < .001).

### 3.2 Females

**FIGURE 8:**
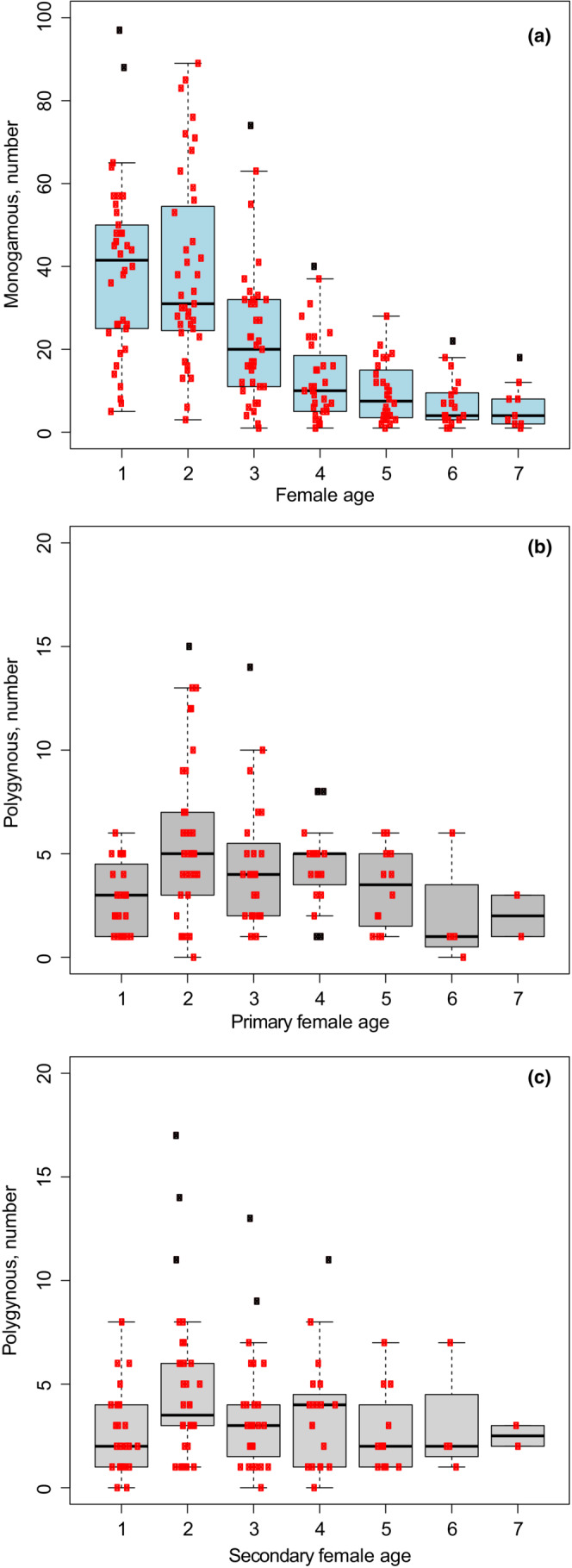
Female age distribution summed per year for mating with monogamous (blue boxes) and polygynous males (gray plots). Primary and secondary females mating with polygamous males are displayed separately. The horizontal line is the median value, the box delimits the 1st and 3rd quartiles [interquartile range (IQR) criterion], while the whiskers (error bars) show the 5th and 95th percentiles. The dots are the data (in gray when outliers).

The primary females included a significantly higher share of older females compared with secondary females and females mated with monogamous males (paired *t*‐test, *p* < .001, Figure 8). While 46% (*n* = 205) of secondary females and 50% (*n* = 1841) of females mated with monogamous males were first‐year breeders, the equivalent percent was 26% (*n* = 208) for primary females. For all age classes above 1‐year old, primary females made up a higher percentage than both secondary females and females of monogamous males.

**FIGURE 9:**
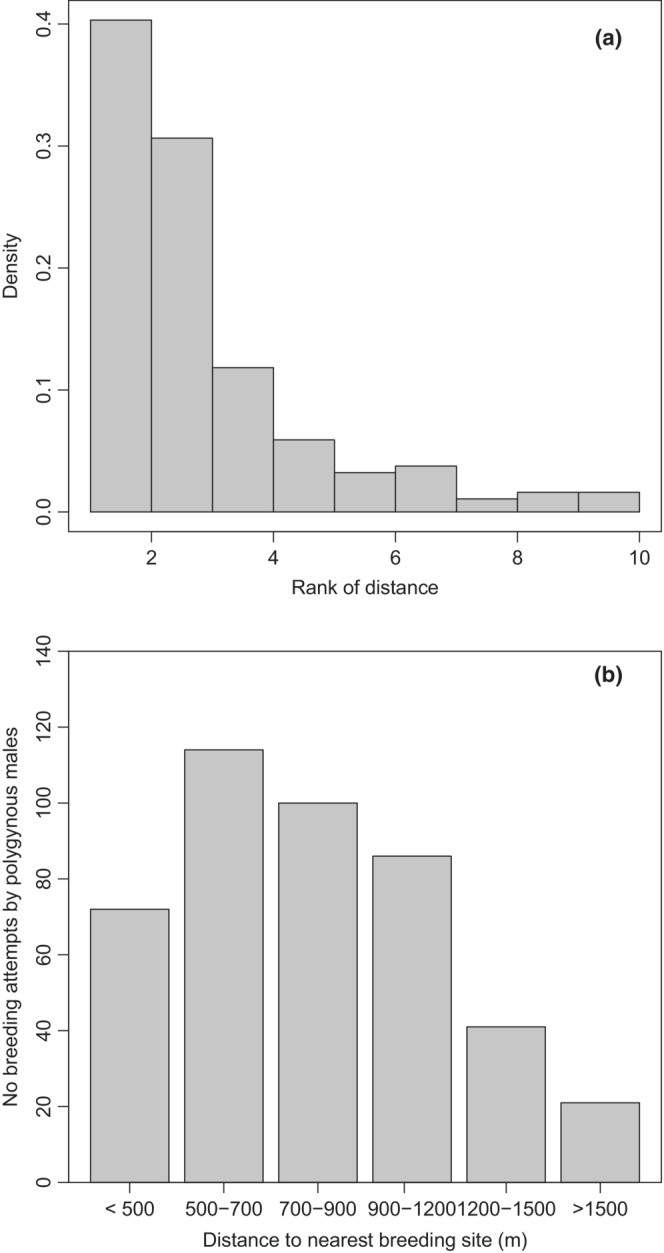
The relative distance of the secondary nest to the primary nest in rank (left, see methods) and in distance (m, right).

A total of 38% of the secondary females were localized in the neighboring breeding site to the primary females, and 29% to the second nearest (Figure 9a). The most commonly recorded distance between nest sites used by a primary and secondary female mated with the same male was 500–700 m (Figure 9b). The most extreme distance between two nesting sites occupied by the same polygynous male was as much as 40 km (S. Almedal, personal communication). Comparing the observed distribution to a uniform distribution with a 1000‐step bootstrap, we found that significantly higher numbers of secondary nests were in the nearest breeding site to the primary nest (*p* < .001).

A small fraction of the females started egg laying already in March, while reproductive onset in April was most common, holding for 75% of the polygynous and 71% of the monogamous males, respectively. Compared with monogamous males, a higher fraction of the polygynous males started breeding with their primary female in the first half of April, while this was reversed over the following 30 days. The median egg‐laying date was 19 April for primary females of polygynous males and 21 April for females of monogamous males (*t*‐test, *p* = .21). Secondary females of polygynous males started breeding 4 days later than those mated to monogamous males (25 April) (*t*‐test, *p* = .01).

In 24 of 25 cases, where the exact onset of egg laying for both the primary and secondary female was known, the difference in time was <23 days, meaning that the primary female was still incubating when the secondary female started egg laying. There were only three cases where the incubation of the two females of a polygynous male started simultaneously, and only one case where hatching had occurred in the primary nest before the secondary female started egg laying. The median interval in onset of egg laying by the primary and secondary female was 7 days (mean = 8.4, SD = 7.2, *n* = 25).

## 4 DISCUSSION

A prevalent hypothesis has been that polygyny is an evolutionary response to unbalanced sex ratios favoring females (i.e., Wittenberger, 1976). However, studies have also shown that polygyny occurs in birds with sex ratios close to unity (Møller, 1986). The mating system of dippers was for long regarded as generally monogamous (i.e., Cramp, 1988) or “irregularly polygynous” (Møller, 1986), but Wilson (1996) found that polygynous males occurred every year in his study population (in total 709 pairs surveyed with irregular intervals in the period 1934–1991). The frequency of polygynous males recorded in our study, 7% involving 15% of all pairs, supports the results of Wilson (1996) and like in his study we also found cases of trigamy. We could only document one territorial male that apparently remained unmated throughout the breeding season.

However, in contrast to Wilson (1996) who found that the frequency of polygynous males differed strongly among years (4%–25%), we found that the fraction of polygyny was fairly stable over the entire study period (Figure 3). Our results do not support the prediction that polygyny is most common at low population density, based on the assumption that at low densities males can defend larger territories with potentially more nest sites.

In the present study, the majority of nest sites were located in the high number of small tributaries along the main river where a restricted part of the main river is included in the territory for foraging. The males assigned to be monogamous may have included a few polygynous males. However, judged from our extensive fieldwork, these are likely to be rare exceptions. Polygynous males normally defended a territory including two breeding sites located within a short distance, which means that if one female was breeding outside the main catchment, this was very likely in a neighboring catchment. As stated previously, we made a substantial effort to detect possible nesting in neighboring catchments. This effort revealed 527 males involved in 830 nesting attempts. However, only five polygynous males were found to have a female outside River Lygna. This confirms that almost all polygynous males had their second nest site within the main catchment.

After having finished a breeding attempt in the lowlands, some males evidently moved to a higher altitude and mated with a new female. Larsson and Tägtström (2012) referred to an example of a banded male dipper that had a second nesting attempt with a new female 472 km apart from his first female. Because most dippers disappear after breeding, and some are recovered breeding at higher altitudes in Lygna or neighboring rivers later in spring, we presume that this pattern is general, that most dippers breed more than once during one breeding season. Anyway, as far as we decided not to assign dipper males with failed nesting and then nesting with a different female (successive bigamists) as polygynous, this insecurity of mating status would not influence our results.

The “popularity” of breeding sites varied from those used more or less every year to those used only once. However, we did not find any difference between polygynous and monogamous males with regard to their ownership of breeding sites ranked according to their overall frequency used. This is surprising because according to the polygyny threshold model, females should only be willing to settle with an already‐mated male if he can provide a high‐quality territory (Orians & Pearson, 1969). The result suggests that the polygynous dippers in our study area had to accept breeding sites of the same quality as monogamous males, to be able to defend more than one. On the other hand, polygynous males were in general older than monogamous ones, supporting results of previous studies of passerine birds (Potti & Montalvo, 1993; Santoro et al., 2022). The oldest breeding males were monogamous, suggesting senescence and life‐history trade‐offs where reproductive effort is counterbalanced by the reduced life span of polygynous males. Possibly the measure of territory quality used here, frequency of use, does not fully reflect inherent habitat quality (Nilsson et al., 2019). It should be noted, however, that older males are represented by few individuals.

The primary females were in general older than females mated to monogamous males. Likewise, Wilson (1996) noted a weak, but non‐significant tendency for primary females to be older on average than secondary females. We found that the age distribution among secondary females followed roughly the same pattern as females mated to monogamous males. An explanation for a higher frequency of older females among primary females may be related to differences among the females at the time of settlement in spring. A larger fraction of the older females may be resident throughout the winter, and if migratory, they may settle earlier in spring than the first‐year birds. They may also be more experienced and know better where to find suitable territories and nest sites along the river. Early settlement and onset of breeding by older females may in turn cause a greater opportunity for the male to defend another territory and engage in more courtship displays to attract a second mate. However, in the present study, polygynous males started breeding marginally earlier than monogamous males.

The majority of primary and secondary females mated in the vicinity of each other. Our data also revealed that there may be a certain minimum distance between nests of primary and secondary females. The optimal distance from the males' perspective may therefore represent a tradeoff between the risk of confrontation between the two females and flying distance. Dippers commonly follow open water, thus the effective distance between the two nests might be considerably longer than the straight line between them, and these figures must therefore be judged with care.

Our data showed that the probability for a polygynous male to perform repeated polygyny over years was significantly higher than by chance. In contrast, Schlicht and Kempenaers (2021) found that of a total of 35 instances of polygyny in the blue tit (*Cyanistes caeruleus*) across 12 years, no male was polygynous in more than 1 year, suggesting high cost. The slightly reduced lifespan in males with repeated polygyny in our study likewise suggests cost in terms of longevity. On the other hand, the cumulative offspring number in these males should still be higher. The literature on repeated polygyny in birds is however scarce. In the collared flycatcher *Ficedula albicollis*, sons of polygynously mated fathers were less likely to become polygynously mated during their lifetime than sons of monogamous males (Gustafsson & Qvarnström, 2006). The quality of the territory might be important, but we found no difference between polygynous and monogamous males with regard to territory quality assessed by their frequency of use.

In conclusion, our long‐term study on mating strategies in the white‐throated dipper confirmed that a significant and quite constant fraction of the males is polygynous, that different age structures exist related to the various mating strategies, and finally that the probability for a male to be involved in polygyny more than once, was significantly higher than by chance.

Our analysis has revealed, based on an unprecedented spatial and temporal dataset in space of time, that polygyny is prevalent in the territorial dipper. Further analyses should include the variation in breeding success among the territories and nest sites, as well as long‐term cost–benefit tradeoffs in the context of polygamy both from the male and female perspective.

## AUTHOR CONTRIBUTIONS

**Bjørn Walseng:** Writing – original draft (lead); writing – review and editing (lead). **Joel M. Durant:** Formal analysis (lead); visualization (lead). **Dag O. Hessen:** Writing – original draft (equal); writing – review and editing (equal). **Kurt Jerstad:** Data curation (lead); funding acquisition (equal); writing – original draft (supporting). **Anna L. K. Nilsson:** Funding acquisition (equal); writing – original draft (equal); writing – review and editing (equal). **Ole W. Røstad:** Data curation (lead); writing – original draft (supporting); writing – review and editing (supporting). **Tore Slagsvold:** Writing – original draft (equal); writing – review and editing (equal).

## Data Availability

The data that support the findings of this study will be openly available in the NIRD Research Data Archive at https://doi.org/10.11582/2022.00047.
